# Use of Zirconia to Restore Severely Worn Dentition: A Case Report

**DOI:** 10.1155/2012/324597

**Published:** 2012-09-05

**Authors:** Manish Agrawal, Banashree Sankeshwari, Channaveer V. Pattanshetti

**Affiliations:** ^1^Department of Prosthodontics and Crown and Bridge, Bharati Vidyapeeth Dental College and Hospital, Sangli 416 416, India; ^2^Department of Oral and Maxillofacial Surgery, Bharati Vidyapeeth Dental College and Hospital, Sangli 416 416, India

## Abstract

The management of tooth wear has been a subject of increasing interest from both preventive and restorative points of view. Severe tooth wear is frequently multifactorial and variable. Successful management is a subject of interest in dentistry. A critical aspect is to determine the occlusal vertical dimension (OVD) and a systematic approach that can lead to a predictable and favorable treatment prognosis. Management of patients with worn dentition is complex and difficult. Accurate clinical and radiographic examinations, a diagnostic wax-up, and determining OVD are crucial. This paper describes the full-mouth rehabilitation of a 47-year-old bruxer with a severely worn dentition.

## 1. Introduction

Restoration of the severely worn dentition is one of the most challenging procedures in dentistry. In order to successfully restore and maintain the teeth, one must gain insight into how the teeth arrived at this state of destruction. Tooth wear can result from abrasion, attrition, and erosion [1–5].

While all occlusions wear to some degree over the lifetime of the patient, normal physiological wear usually does not require correction [6]. Severe or excessive wear refers to tooth destruction that requires restorative intervention. Severe attritional wear can result from occlusal prematurities that prevent functional or parafunctional movements of the jaw.

Evaluation and diagnosis should account for the patient's diet, history of eating, and/or gastric disorders, along with the present state of the occlusion. Emphasis must be placed on the evaluation of occlusal prematurities preventing condylar seating into the centric relation position [6]. Behavioural factors that may contribute to parafunctional habits and/or nocturnal bruxism are also important to understand and manage in order to successfully restore and maintain a healthier dentition [7]. Once a complete understanding of the etiology of the dentition's present state is appreciated, a treatment plan can be formulated, taking into account the number of teeth to be treated, condylar position, space availability, the vertical dimension of occlusion (VDO), and the choice of restorative material [8].

Full-mouth rehabilitation is the combination of all the different dental specialties under one treatment plan, covering all different dental aspects under one umbrella. Dentistry is a quickly evolving field. New treatments are being created while the old ones are being perfected. Smile makeovers are becoming more common with dental veneers and/or metal-less porcelain crowns. New materials are constantly improving the dental procedures, enhancing the patients comfort. In summation, we could say that full mouth rehabilitation is the combination of dental occlusion, dental balance, and functionality, with dental esthetics.

This paper describes the stages in restoration of severely worn dentition due to bruxism. The steps in treatment of these patients include a comprehensive examination, diagnostic mounting and diagnostic wax-up, careful planning and sequencing of various steps, discussion with the patient about the different treatment alternatives, and careful execution of the treatment plan.

## 2. Case Report

**Figure 1:**
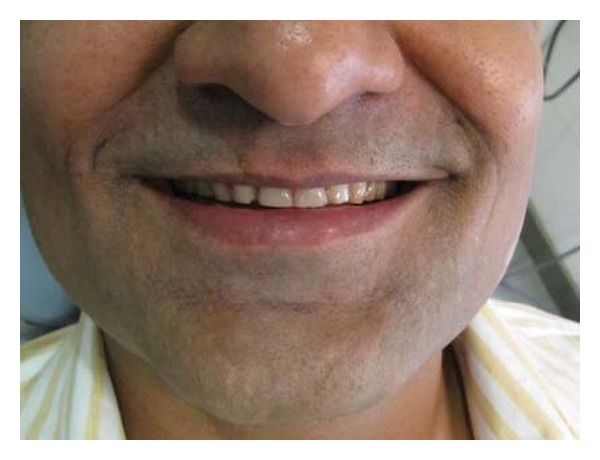
Preoperative extraoral view.

**Figure 2:**
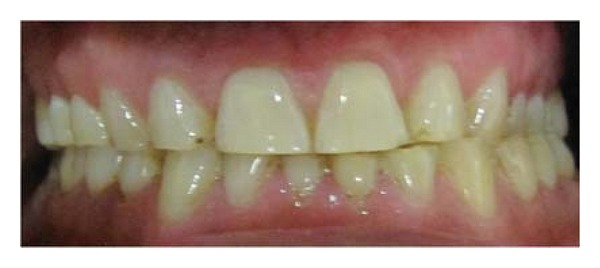
Preoperative intraoral view.

A 47-year-old male patient reported with the chief complaint of chronic sensitivity with anterior and posterior teeth. Clinical examination revealed severely worn anterior and posterior teeth (Figures 1 and 2). On evaluation and diagnosis, patient's behavioural history related to stress which may have led to parafunctional habits. Endodontic and periodontal status was good except for mandibular left and right central and lateral incisors which needed endodontic treatment. Evaluation of the TMJs was unremarkable, with normal jaw opening and range of motion. No joint sounds, signs or symptoms of instability were evident. Joint loading in centric relation was performed utilizing bimanual manipulation.

Clinical investigation like phonetics—the S sound altered to F due to increased space, signs of wear, interocclusal distance was increased due to wear than normal (2–4 mm), facial appearance showed wrinkles, and drooping commissures at the corner of the mouth indicated loss of vertical dimension.

The findings were explained to the patient, and treatment options were presented. The treatment goals were to restore the lost occlusal vertical dimension (OVD) to correct the occlusal plane, to restore function, and to restore the esthetics of the patient's dentition.

**Figure 3:**
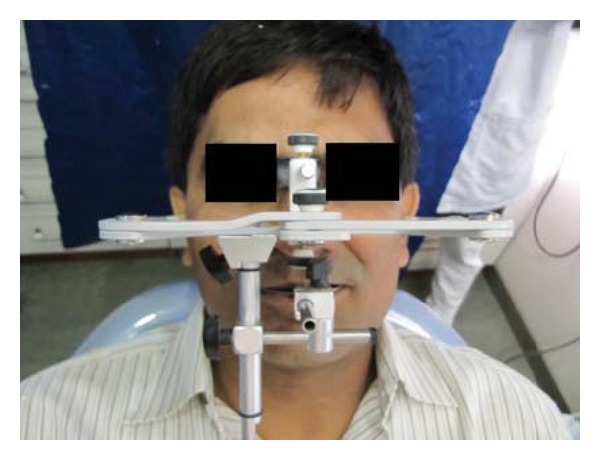
Facebow transfer.

**Figure 4:**
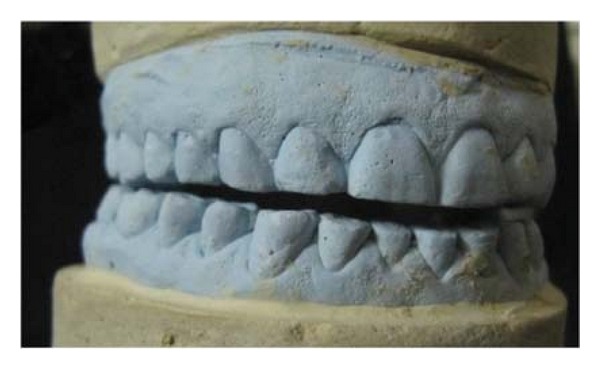
Restoration of vertical dimension on articulator.

**Figure 5:**
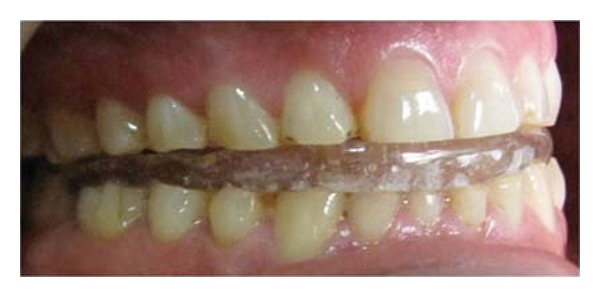
Occlusal splint in place.

Impressions for study casts were made with irreversible hydrocolloid (Zelgan 2002, Dentsply) material along with a centric relation occlusal record. The patient's casts were mounted on a semiadjustable articulator (Bio-art model A7, Brazil) using facebow record and the centric relation record (Figure 3). The new VDO was set by 3-4 mm increase in incisal guidance pin of the articulator (Figure 4). Occlusal splint was fabricated with the new VD and was designed to offer bilateral contacts of posterior teeth in centric relation (Figure 5). The anterior guidance disoccluded the posterior teeth except in centric relation. The adaptation of the patient to increased VD was evaluated during 6-week period. No muscle tenderness or discomfort in TMJ was noted.

**Figure 6:**
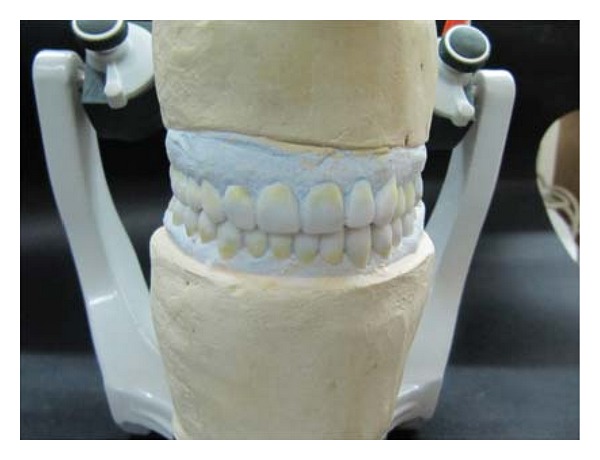
Diagnostic wax-up.

 Diagnostic wax-up was performed at the restored VD (Figure 6). It was then followed by prosthetic phase.

Replacing the anteriors and harmonizing the anterior guidance forms the first step in treatment. The mandibular anteriors were prepared to receive all ceramic crowns, gingival displacement was done. Impressions were made with single-step putty-wash technique using addition silicone material (Aquasil, Dentsply, Germany) and casts poured using type IV dental stone (Kalrock, Kalabhai, India). The anterior guidance was established using anterior plane and esthetics as guide. Provisional restorations were fabricated for the mandibular anteriors with autopolymerizing resin using the putty index and were reshaped in the mouth to achieve ideal contour and cemented using eugenol free temporary luting cement (Rely X Temp NE, 3 M ESPE, Germany).

The anterior replacement was followed by management of the posteriors. A putty index was fabricated for the wax-up in each quadrant which served not only as a guide for the tooth preparation in each quadrant but also in the fabrication of provisional restoration.

**Figure 7:**
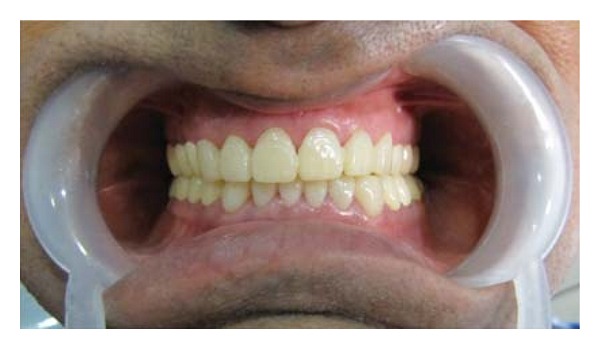
Provisional restorations were placed.

The posterior quadrants were prepared for full-coverage metal ceramic crowns. Mandibular posteriors were prepared followed by the maxillary posteriors. Gingival displacement was done and impression was made with single-step putty-wash technique using additional silicone material. The working casts were articulated using facebow transfer and interocclusal centric record at previously determined vertical dimension. The provisional restorations were fabricated using putty index by the indirect technique, it was finished and corrected for occlusal interferences and luted with eugenol free temporary luting cement (Figure 7). 

Patient's adaptation was monitored for a period of 6 weeks. The patient was assessed for adequate esthetics and functional harmony. 

**Figure 8:**
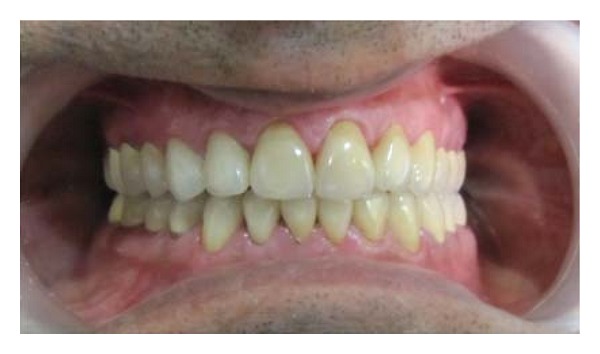
Definitive restoration.

Once the occlusal adjustments, speech and esthetics seemed satisfactory, an occlusal bite record was taken. Definitive impressions were made with additional polyvinyl siloxane (Aquasil, Dentsply, Germany). Casts were mounted on the articulator with facebow transfer and the occlusal bite record in centric and eccentric relations. The final restorations were fabricated and cemented with FugiCEM cement (Figure 8). The scheme of occlusion given to the patient was mutually protected occlusion.

Patient was evaluated for 24 hrs and 48 hrs and necessary occlusal corrections were done. The patient was instructed oral hygiene maintenance and was advised six monthly check-up. A protective splint was fabricated to prevent further damage to the restorations.

## 3. Discussion

The etiology of occlusal wear for our patient is not fully understood; however, it can be hypothesized that the patient had history of stress which may have led to parafunctional occlusal habit and started grinding his anterior teeth. Once the anterior teeth got shorter, the patient lost anterior guidance and developed posterior interferences. The posterior interferences in lateral excursions can activate the masseter and temporalis muscles, enabling the patient to generate more forces to grind his teeth more aggressively [9]. A mutually protected occlusal scheme was used to prevent the destruction of the new prostheses.

Mutually protected articulation is described as “an occlusal scheme in which the posterior teeth prevent excessive contact of the anterior teeth in maximum intercuspation, and the anterior teeth disengage the posterior teeth in all mandibular excursive movements.” Studies have shown that in lateral excursive movements [10], the anterior teeth can best receive and dissipate the forces [11] and posterior contacts in excursions appear to provide unfavorable forces to the masticatory system because of the amount and direction of the applied forces [12, 13].In addition to the use of the mutually protected occlusal scheme, an occlusal splint was fabricated for night wear, and the patient was instructed and trained to keep his teeth apart when not actively chewing.

Zirconia ceramic crowns were selected for the patient's treatment to ensure adequate strength on upper and lower anterior teeth. The primary advantage of a zirconia restoration is esthetic benefit as it is translucent and tooth-colored.A metal-free ceramic crown can transmit a great amount of incident light through to a ceramic core where light is scattered in a natural fashion [14].Thus, the appearance of definitive restorations may be very close to that of a natural tooth.

Many factors influence the choice of all-ceramic systems for abraded upper incisors. Strength, fit, and esthetics are traditionally considered in the selection of material for full-coverage restorations. Anterior crowns can be fixed to the tooth by traditional cements or resin cements. Traditional cements occupy the space between the restoration and the tooth surfaces but do not provide adhesion between them. Resin cements provide adhesion to both surfaces and can act to transfer force from the restoration to the underlying tooth and strengthen all-ceramic restorations [15].

A protective splint with a medium thermoplastic sheet was fabricated and given to the patient to prevent further tooth damage due to bruxism.

## 4. Conclusion

Full mouth rehabilitation is a treatment modality which not only focuses on the esthetics and functional aspect of the dentition but also improves upon the health of the whole stomatognathic system. A detailed diagnosis and treatment planning is necessary to achieve predictable success.
